# Reprogramming of Fatty Acid Metabolism in Gynaecological Cancers: Is There a Role for Oestradiol?

**DOI:** 10.3390/metabo12040350

**Published:** 2022-04-14

**Authors:** Azilleo Kristo Mozihim, Ivy Chung, Nur Akmarina B. M. Said, Amira Hajirah Abd Jamil

**Affiliations:** 1 Department of Pharmaceutical Life Sciences, Faculty of Pharmacy, University of Malaya, Kuala Lumpur 50603, Malaysia; mgn180036@siswa.um.edu.my (A.K.M.); nur_akmarina@um.edu.my (N.A.B.M.S.); 2 Department of Pharmacology, Faculty of Medicine, University of Malaya, Kuala Lumpur 50603, Malaysia; ivychung@ummc.edu.my

**Keywords:** fatty acid, obesity, gynaecological cancer, cancer metabolism, oestradiol

## Abstract

Gynaecological cancers are among the leading causes of cancer-related death among women worldwide. Cancer cells undergo metabolic reprogramming to sustain the production of energy and macromolecules required for cell growth, division and survival. Emerging evidence has provided significant insights into the integral role of fatty acids on tumourigenesis, but the metabolic role of high endogenous oestrogen levels and increased gynaecological cancer risks, notably in obesity, is less understood. This is becoming a renewed research interest, given the recently established association between obesity and incidence of many gynaecological cancers, including breast, ovarian, cervical and endometrial cancers. This review article, hence, comprehensively discusses how FA metabolism is altered in these gynaecological cancers, highlighting the emerging role of oestradiol on the actions of key regulatory enzymes of lipid metabolism, either directly through its classical ER pathways, or indirectly via the IGIFR pathway. Given the dramatic rise in obesity and parallel increase in the prevalence of gynaecological cancers among premenopausal women, further clarifications of the complex mechanisms underpinning gynaecological cancers are needed to inform future prevention efforts. Hence, in our review, we also highlight opportunities where metabolic dependencies can be exploited as viable therapeutic targets for these hormone-responsive cancers.

## 1. Introduction

Gynaecological cancers are one of the leading cancers afflicting women, accounting for almost 40% of new cancer cases and approximately one in three cancer deaths in 2020 among women [1]. More worryingly, these cancers have shown an increase in both prevalence and mortality rate over the past decades, although specific epidemiological patterns vary, depending on various factors, such as geographical location, ethnicity, and socioeconomic status [2,3,4]. Specifically, breast cancer remains the leading cause of cancer-related deaths among women, while ovarian, cervical and endometrial cancers are among the top 10 cancer types affecting women globally [1].

Cancer cells undergo metabolic reprogramming to support cell proliferation, growth, and dissemination, a trait now considered a hallmark of cancer. In doing so, similar to metabolically active cells in the heart [5], cancer cells can metabolically switch to confer themselves with increased nutrient uptake and energy supply, even under nutrient-stress conditions [6,7]. Alterations in lipid metabolism, and specifically, the uptake and synthesis of fatty acids (FAs), constitute one well-documented aspect of this reprogramming. Fatty acids (FAs) are carboxylic acids, consisting of hydrocarbon chains with varying degrees of length, branching and saturation. They act as primary building blocks for lipid species, such as phospholipids, sphingolipids and triglycerides, all of which participate in a wide array of biological processes [8]. In addition to these roles, FAs are also well established as having a critical role in altering gene transcription by regulating the activity of FA-sensitive transcription factors, particularly sterol-regulatory element-binding protein (SREBP) and peroxisome proliferator-activated receptors (PPARs) [9]. More recently, dysregulated FA metabolism has been reported and studied in many cancer types, including gynaecological cancers [10,11,12]. FA metabolism supports tumorigenesis and cancer progression through a range of processes, including membrane biosynthesis, energy storage and production, and generation of signalling intermediates [11,12].

Female sex hormones, specifically oestradiol, play a crucial role in regulating FA metabolism and are also implicated in promoting the risk of gynaecological cancers. The increased risk for these cancers and their pathogenesis has been epidemiologically linked to abnormally high levels of serum oestradiol [13,14,15,16,17,18]. Supporting this, shorter duration of oestradiol exposure is inversely related to the risk for these cancers [19,20,21]. The risk for endometrial cancer, for example, is lowered by 4% for every 2 years of delay in the age of menarche [22]. The lower risk for gynaecological cancers is also contributed by periods of lower serum oestradiol levels, such as during breastfeeding, with the risk being greatly reduced the longer the breastfeeding duration [23,24,25]. Emerging evidence indicates aberrant FA metabolism is postulated to be mediated by the action of oestradiol, either directly via their classical, oestrogen receptor (ER)-mediated pathways, or indirectly through the insulin-like growth factor I (IGFI) receptors (IGIFR), with the levels of serum oestradiol and the IGFIR pathway both dysregulated, not only in gynaecological cancers but also in obesity. This might explain the significant role obesity plays in increasing the risk and promoting the malignancy of gynaecological cancers among obese patients [26,27]. Indeed, it is now well established that the relative risks of breast, ovarian, cervical and endometrial cancer positively correlate with increasing Body Mass Index (BMI), one of the measures of obesity [28].

Decades of research have provided tremendous insights into the integral role of FAs in tumourigenesis, but the metabolic role of high endogenous oestrogen levels and increased gynaecological cancer risks, notably in obesity, is less understood. In addition, the molecular players involved in the oestradiol-driven alteration of FA metabolism across gynaecological cancer is still underexplored. This review article, hence, comprehensively discusses how FA metabolism is dysregulated in gynaecological cancer and the potential role of oestradiol in this process, highlighting the emerging role of oestradiol in affecting the actions of key regulatory enzymes of lipid metabolism, either directly through its classical ER pathways or indirectly via the IGFIR pathway. We also explore how the actions of oestradiol on FA metabolism could be altered with obesity, as increasing BMI is intricately linked with increased risk and poorer a prognosis in gynaecological cancers. This review aims to further clarify our understanding of the role of oestradiol in altering the FA metabolism in gynaecological cancers, providing opportunities to therapeutically target metabolic dependencies in hormonal responsive gynaecological cancers, particularly in the context where therapies targeting the oestradiol pathway are also used.

## 2. Methods

This review includes studies that investigated the dysregulation of key enzymes involved in four FA metabolic pathways (i.e., uptake, de novo synthesis, activation, and oxidation) in gynaecological cancers (i.e., breast, ovarian, cervical, and endometrial cancer). Epidemiological, clinical, and molecular studies on how oestradiol could influence FA metabolism in gynaecological cancers in both obese and non-obese conditions were also included, along with studies that explored pharmacological and dietary interventions metabolically targeting these cancers. Study searches were performed using the PubMed database from the National Library of Medicine, National Institutes of Health, Web of Science database by Clarivate and clinical trials from www.cancer.gov-US (accessed from March 2021 to March 2022), among others.

## 3. Altered Fatty Acid Metabolism in Gynaecological Cancers

FAs are a family of lipids, of which the structure consists of two main components: (1) a straight chain of carbon atoms and (2) a carboxylic group [29]. The length of the straight chain can vary enormously, ranging from 2 to 36 carbon atoms. On top of this chain-length variation, the chain can also be either fully saturated (e.g., palmitic, stearic and arachidic acid), where it contains only carbon–carbon single bonds or desaturated where it also has carbon–carbon double bonds. Desaturated FAs can be further classified into two groups based on the number of carbon–carbon double bonds present in the chain: monounsaturated FAs (e.g., myristoleic, palmitoleic and elaidic acid), which have only one carbon–carbon double bond, and polyunsaturated FAs (e.g., linoleic, stearidonic and docosahexaenoic acid (DHA)), which have more than one [8].

**Figure 1:**
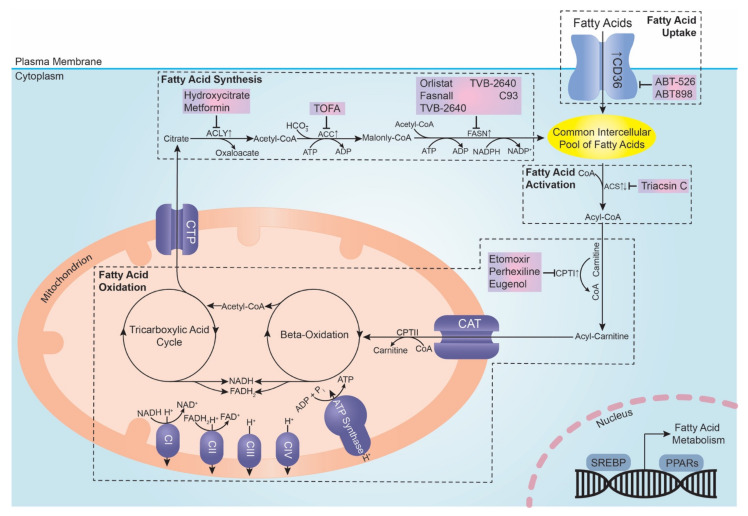
Overview of the various aspects of fatty acid metabolism and their dysregulation in gynaecological cancers. Fatty acids can be produced via de novo synthesis using citrate-derived acetyl-CoA or obtained exogenously through CD36. They are then activated to produce acyl-CoAs which are then transported into the mitochondrion to undergo beta-oxidation, producing NADH, FADH2 and acetyl-CoA all of which are used to synthesise ATP via the TCA cycle and ETC. Fatty acid metabolism is mainly regulated by the transcription factors SREBP and PPARs. An upwards arrow (↑) indicates an overall trend of upregulation of an enzyme across gynaecological cancer types whereas an upward–downward arrow pair (↑↓) indicates variable expression pattern depending on cancer type and enzyme isoform. Pharmacological inhibitors have been developed for each FA metabolism enzyme highlighted here. CTP, Citrate transport protein; ACLY, ATP-citrate lyase; ACC, acetyl-CoA carboxylase; FASN, Fatty acid synthase; ACS, Acyl-CoA synthetase; CPTI, Carnitine palmitoyltransferase I; CAT, Carnitine-acylcarnitine translocase; CPTII, Carnitine palmitoyltransferase II; TCA, Tricarboxylic acid; CI, Complex I; CII, Complex II; CIII, Complex III; CIV, Complex IV; SREBP, sterol regulatory element-binding proteins; PPAR, peroxisome proliferator-activated receptor.

In mammalian cells, FAs can either be obtained through direct exogenous uptake from the surrounding microenvironment or synthesised de novo from nutrients, such as glucose and glutamine. It is widely accepted that lipidomic remodelling is a metabolic hallmark of cancer cells, including gynaecological cancers. This remodelling broadly encompasses alterations in the following four processes: (1) FA transport/uptake, (2) de novo FA synthesis, (3) FA activation and (4) FA oxidation to generate ATP (Figure 1). The specific mechanisms driving particular lipid phenotypes are nuanced and may be dependent on tumour types or molecular sub-classifications.

### 3.1. Fatty Acid Uptake

One way cells acquire FAs is through the uptake of exogenous FAs (Figure 1), facilitated by FA transport proteins, such as the members of the FATP family (FATP1-6) [30] and CD36. FA uptake plays a key role in allowing cancer cells to metabolically adapt and progress in specific environmental niches that have an abundance of adipocytes to act as a source of exogenous FAs [31,32]. While a variety of plasma membrane proteins are found to facilitate exogenous FA uptake, CD36 is the only one so far demonstrated to act as a genuine FA transporter.

#### CD36

CD36 is a ubiquitous membrane glycoprotein shown to be a genuine FA transporter possessing an FA-binding pocket and a cavity serving as a tunnel for FA transfer [33]. Altered CD36 expression is reported in breast [34] and cervical [35] cancers (Table 1), with many experimental findings strongly suggesting CD36 to be a key player in promoting gynaecological cancer progression via its role as an FA uptake protein. The understanding of the FA uptake role of CD36 in the progression of these cancers has been greatly substantiated by loss-of-function studies and adipocyte co-culturing. Depleting CD36 in MCF-7 and HeLa cells treated with oleic acid reversed oleic-acid-induced proliferation, migration, and invasion [34,35]. This cancer phenotype reversal is largely attributed to the inhibition of CD36-mediated FA uptake as signified by the reduced formation of lipid droplets. In line with this, chemically inhibiting and ablating CD36 reduced the uptake of adipocyte-derived FA in ovarian cancer cells co-cultured with adipocytes, inhibiting tumorigenic potential and metastasis [31]. Although CD36 is yet to be shown to be involved in the progression of endometrial cancer, FA uptake may still be crucial in its development. Phloretin, an FA uptake inhibitor, has been shown to reduce the cell viability of various endometrial cancer cell lines [36], Collectively, these findings demonstrate that the FA uptake function of CD36 plays a key role in mediating the uptake of exogenous FAs in gynaecological cancers, contributing to their malignancy. However, it should be borne in mind that CD36 is a multifunctional protein with both pro-tumorigenic and anti-tumorigenic properties [37]. Indeed, an inverse relationship has also been found between CD36 expression levels and aggressiveness: more aggressive breast cancer cell lines such as MDA-MB-231 have lower CD36 expression levels than that of less aggressive ones, such as MCF-7 [38]. Such a discrepancy highlights the potential of multifunctional proteins to exhibit starkly opposing behaviours, depending on the extracellular milieu. In the studies showing a positive relationship between CD36 and malignancy, exogenous FAs were abundantly provided and cancer cells upregulated CD36 expression for FA uptake to promote aggressiveness. In contrast, in studies showing a negative relationship, exogenous FAs were not as abundantly supplied, and cancer cells downregulated CD36 expression. This decreased CD36 expression might stem from the lack of pro-tumorigenic CD36 activities (i.e., exogenous FA uptake) to offset anti-tumorigenic ones (i.e., anti-angiogenesis and apoptosis) [37] in an environment devoid of substantial exogenous FAs. Yet, it is intriguing to observe high expression of CD36 in tamoxifen-resistant MCF-7 breast cancer cells and its inhibition led to restoration of tamoxifen-mediated growth inhibition [39]. Thus, this points to the possibility of stratifying breast cancer patients based on CD36 expression to determine their suitability for tamoxifen-based chemotherapy. Nevertheless, whether this CD36-mediated resistance against tamoxifen can be extended to other chemotherapeutic drugs and stems from the specific role of CD36 in FA uptake warrants further study.

### 3.2. De Novo Fatty Acid Synthesis

A variety of FAs are endogenously produced in the cells, both saturated (e.g., palmitic acid and stearic acid) and unsaturated (e.g., palmitoleic acid and oleic acid) [64]. FAs are synthesised from acetyl-CoA transported in the form of citrate from the mitochondria to the cytosol. Once in the cytosol, citrate is cleaved by cytoplasmic ATP citrate lyase (ACLY) to release acetyl-CoA, which is then converted to malonyl-CoA by acetyl-CoA carboxylase (ACC). Subsequently, malonyl-CoA is utilised by fatty acid synthase (FASN) to produce a 16-carbon-saturated FA palmitate via the sequential addition of two-carbon units derived from acetyl-CoA via a repeated series of condensation, reduction and dehydration reactions [65] (Figure 1). Palmitate can undergo subsequent reactions, such as elongation and desaturation to supply the cells with a wide variety of lipids, including triacylglycerols [66]. The expression levels of FA-synthesising enzymes are controlled by the sterol regulatory element-binding proteins (SREBP) family of transcription factors with the isoform SREBP-1c being primarily involved in regulating FA-synthesising enzymes [67]. 

De novo FA synthesis is augmented in cancer cells, regardless of the availability of exogenous FAs [68], in contrast to normal cells, which prefer to acquire FA exogenously, except for a few cell types, such as adipocytes and hepatocytes [69]. The extent of FA synthesis and higher tumour grade are strongly correlated, highlighting their potential use as a clinical marker for tumour aggressiveness [70,71,72]. This strong correlation primarily stems from the multifaceted role played by FA in the many processes involved in tumour progression, such as immune evasion, angiogenesis and oxidative resistance [73]. Therefore, it is unsurprising that FASN, the enzyme directly synthesising FA, is the most focused on thus far (Figure 1). However, aside from FASN, other enzymes involved in de novo FA synthesis also play an instrumental part in tumour progression, such as ACC, the rate-limiting enzyme for de novo FA synthesis, and ACLY, which provides glucose-derived acetyl-CoA as the main substrate for both ACC and FASN (Figure 1).

#### 3.2.1. ATP Citrate Lyase

ATP citrate lyase (ACLY) catalyses the breakdown of acetyl-CoA and oxaloacetate from citrate and CoA with the concomitant hydrolysis of ATP to ADP and phosphate [74], with the acetyl-CoA being subsequently used for de novo FA synthesis. Significant evidence suggests an important role for ACLY in the biology of gynaecological cancers, including increased ACLY expression evident in breast [75,76], ovarian [41] and cervical [42] (Table 1) cancers, parallel to the high rate of de novo FA synthesis reported therein. Knocking down ACLY expression in MCF-7 [75] and A2780 [41] cells suppressed their cell viability and inhibited their proliferation. The pro-tumorigenic activity of ACLY is regulated by a variety of post-translation mechanisms, including phosphorylation. Phosphorylated ACLY Ser-455 is higher in breast cancer tissues than in normal ones. The use of ACL Ser-455 phosphomimetic, along with non-phosphorylatable mutants, showed ACLY phosphorylation is crucial in the survival of ACL-depleted MDA-MB-453 cells. Ser-455 ACLY phosphorylation was abrogated after siRNA-mediated depletion of mTORC2 in MDA-MB-361 and MDA-MB-453. This abrogation significantly suppressed their growth, highlighting the pivotal role of mTORC2-mediated phosphorylation of ACLY in breast cancer progression [77]. Interestingly, the metabolic role of ACLY may be linked to cell cycle dysregulation by its binding to low-molecular-weight (LMW) cyclin E, a tumour-specific isoform of cyclin E, to enhance ACLY function [78]. 

#### 3.2.2. Acetyl-Coenzyme A Carboxylase

Acetyl-coenzyme A carboxylase (ACC) produces malonyl-CoA by carboxylating acetyl-CoA and is regulated through a variety of post-translational modifications, including inhibitory phosphorylation [79]. ACC is implicated in the pathology of gynaecological cancers, as shown by the increased ACC expression and activity in breast [46] and ovarian [80] cancers (Table 1). Different genetic variants of ACC also play a role modifying cancer risks. Certain ACCα haplotypes were found associated with significantly increased breast cancer risk, while several others were found linked to reduced risk [81]. Inhibiting ACC via siRNA in breast cancer cells led to cell growth and apoptosis in several breast cancer cell lines [44]. Inhibiting ACC using 5-(tetradecyloxy)-2-furoic acid (TOFA) in ovarian cancer cell lines was found to reduce cell proliferation, arrest cells in G0/G1 and induce apoptosis in vitro and in vivo [45]. However, in TOFA-treated breast cancer cell lines, a lack of growth inhibition is found [82]. In some cases, inhibition of ACC reduces cell growth only when there is concurrent inhibition of another FA metabolism enzyme. In breast cancer cells, TOFA alone in some instances did not inhibit cell growth, while pairing it with the CPTI inhibitor etoxomir did [83]. This discrepancy suggests ACC may not be as crucial of an enzyme when it comes to cell growth in certain cases. In fact, ACC may not only be unnecessary in some situations, but may also be a hindrance to aggressiveness in particular subtypes of gynaecological cancers; ACC is more downregulated in TNBC than in RP [84]. Nevertheless, ACC might still prove useful in a clinical setting, including predicting the prognosis of patients undergoing certain anti-cancer treatment, and this could be the case for ovarian cancer. In the MITO phase III trial, overexpression of the phosphorylated form of ACC (pACC) was found to predict poor outcomes for ovarian cancer patients receiving carboplatin/paclitaxel [85]. Such prediction reveals the usefulness of pACC expression as a potential prognostic marker, especially for ovarian cancer patients taking carboplatin/paclitaxel [86].

#### 3.2.3. Fatty Acid Synthetase

Fatty acid synthase (FASN) is a multi-subunit enzyme complex, facilitating the successive addition of two-carbon units derived from acetyl-CoA to malonyl-CoA via a repeated series of reactions, involving condensation, dehydration and reduction, to produce palmitate [87]. Evidence consistently suggests a pro-tumorigenic role for FASN in gynaecological cancers. FASN expression is mostly correlated with tumour characteristics and clinical prognosis of gynaecological cancer patients. Higher FASN expression was observed to be linked to higher pathological stages and poorer patient prognosis in breast [88,89,90], ovarian [50,91], cervical [52,53] and endometrial [92,93,94,95] cancers (Table 1). Although FASN is often highly expressed in these cancers, the FASN expression profile exhibits subtype-specific heterogeneity. For example, FASN expression was lower in luminal A and TNBC subtypes than in their healthy counterparts [84,96]. FASN overexpression is also linked to certain molecular signatures, as overexpression was found to be significantly associated with HER2 positivity [90,97]. Interestingly, FASN expression patterns in gynaecological cancers can also differ between the primary and metastatic tumours. Some metastatic tumours have significantly lower FASN expression than their corresponding primary tumours, allowing them to metabolically adapt to the different environments at their respective metastatic site, probably different from that of their primary site. A case in poInt. is in breast cancer [96], where the highest FASN expression was found in brain metastases to accommodate the elevated acetate levels produced by upregulated acetyl-CoA synthetase 2 (ACSS2) for FA synthesis [98], whereas the lowest was found in liver metastases, to shift the metabolic phenotype of metastasising cells to become more glycolytic for overcoming the varying hypoxic barriers present in the liver [99]. The divergence in FASN expression between the different types of metastatic tumours could be one of the clinical criteria in deciding whether to treat breast-cancer-metastasised patients using FASN inhibitors based on the anatomical location of the metastasis. FASN expression in endometrial cancer was also found to gradually increase as the endometrium advances from hyperplasia to carcinoma, suggesting the use of FASN as one of the molecular markers for histological staging [94]. However, Rahman et al., found FASN expression in endometrial cancer was favourably linked to overall patient survival rate [100]. This discrepancy is likely due to their use of only endometrioid adenocarcinoma, whereas the other investigators included other types of endometrial cancer, such as serous and clear cell carcinoma. Such cases warrant caution in using FASN as a potential prognostic marker by considering the specific type of endometrial cancer. Reducing FASN activity retarded the malignancy of several gynaecological cancers, including breast [47,82,90,101,102,103,104,105], ovarian [48,51] and endometrial [100] cancer, in experimental studies using various FASN-inhibiting agents (e.g., C75, orlistat and cerulenin) and genetic perturbations, highlighting the crucial role of FASN in promoting the progression of these cancer types. Moreover, inhibiting FASN using cerulenin-sensitised cisplatin-resistant ovarian cancer cell lines to cisplatin [48] implicates FASN in promoting chemoresistance in gynaecological cancers. FASN can also have an immunoregulatory role to support the immune avoidance in these cancer types. In ovarian cancer cells, constitutively activating FASN compromised the capacity of tumour-infiltrating DCs (TIDCs) to support antitumour T cells and inhibiting FASN restored this capacity [48].

### 3.3. Fatty Acid Activation

Whether exogenously acquired or synthesised de novo, FAs must be activated before they can be processed by both anabolic and catabolic downstream pathways (Figure 1). In many cancer types, studies have demonstrated FA activation is highly dysregulated and largely plays an oncogenic role by promoting various cancer phenotypes, such as cell proliferation [106,107,108], apoptosis evasion [106,108] and invasion [107]. This step is catalysed acyl-CoA synthetase (ACS), a family of enzymes that convert FAs to their respective acyl-CoA form, with the usage of acetyl-CoA and the equivalent of two high-energy bonds (Figure 1). The family consists of 26 members, and these can be further divided based on and named after the chain length of their preferred FA substrate [109].

#### Acyl-CoA Synthetase

Acyl-CoA synthetase (ACS) activates FA by converting free FAs to their corresponding fatty acyl-CoA. Its expression is altered in several gynaecological cancers, including breast [55,56,58] and ovarian [58] cancer (Table 1). Although a general pattern of upregulation is observed, the exact ACS expression profile depends on the specific ACSs, which are tissue and cancer dependent. Specific ACS isoforms, for example, ACSL3 and ACSL5, are both downregulated in ovarian cancer [58]. Quadruple-negative breast cancer tissues, on the other hand, have higher expression of ACSL4 than their healthy counterparts [54], whereas ER- breast cancer overexpresses ACSL1, ACSL3, ACSL4 and ACSL4 [55,56,58]. Functional studies have given mixed results as to the role of certain ACSs in promoting the progression of gynaecological cancers. In breast cancer cells, ACSL4 is required for the cellular uptake of polyunsaturated fatty acids (PUFA) [107] and is implicated in promoting breast cancer phenotypes, such as migration and invasion [54,57]. In contrast, knocking down ACSL4 in MCF-7 did not inhibit cell proliferation [110]. However, this discrepancy could have arisen from the use of different cancer subtypes among the different studies, indicating a subtype-specific role of ACSL4 related to breast cancer progression. In addition, shRNA-knockdown of ACSL4 in MDA-MB-231 increased the inhibitory effect of cisplatin, doxorubicin and paclitaxel, suggesting a role of ACSL4 in promoting chemoresistance [111]. The expression of specific ACS types could also serve as a potential prognostic marker. The high expression of ACLS5 is associated with favourable prognosis in both ER- and ER+ breast cancer patients [56,58]. Homozygous deletion of ACSL3 in Korean TNBC patients, discovered via targeted exome sequencing, found the deletion to be associated with the increased risk of relapse and promoted distant metastasis in breast cancer patients, following adjunct chemotherapy treatment [112]. Similarly, higher expressions of ASCL3 and ASCL5 are associated with better prognosis in ovarian cancer patients [58]. These studies appear to suggest both ACSL3 and ACSL5 probably play a role in hindering cancer progression across different gynaecological cancers. However, downregulation of ACSL3 also inhibited the proliferation, migration, and invasion of breast cancer cell lines [113], alternatively suggesting instead a pro-tumorigenic role. Therefore, the disparate biological outcomes in deleting or downregulating different ACS types in the pathology of gynaecological cancers must be firstly resolved, before pursuing any of the ACS isoforms as potential prognostic markers. 

### 3.4. Fatty Acid Oxidation

After FA’s activation to form fatty acyl-CoA, it must be transported across the mitochondrial membrane, accomplished by first transferring the acyl group from CoA to carnitine, catalysed by carnitine palmitoyltransferase I (CPTI), found embedded on the outer mitochondrial membrane. The resulting acylcarnitine is then transported into the mitochondrial matrix by carnitine-acylcarnitine translocase (CAT), in exchange for free carnitine. In the mitochondrial matrix, the acyl group is transferred back from carnitine to CoA by carnitine palmitoyltransferase II (CPTII), reforming acyl-CoA. The acyl-CoA is then degraded via β-oxidation, a repetition of a four-reaction sequence, with each repetition yielding acetyl-CoA, NADH and FADH_2_, all of which are fed into the Krebs cycle and the electron transport chain (ETC). Various transcriptional and translational mechanisms affect the concentration of β-oxidation enzymes, such as medium-chain acyl-coenzyme A dehydrogenase (MCAD), thereby also regulating the rate of FA metabolism [114]. Once fed into the ETC, NADH and FADH_2_ donate electrons that flow from complexes I to IV of the ETC down an energy gradient. This gradient drives the phosphorylation of ADP to synthesise ATP through the flowing of protons back into the mitochondrial matrix from the intermembrane mitochondrial space via ATP synthase (Figure 1). 

The role of FA oxidation in ATP production is particularly important in cell survival under metabolic stress, such as hypoxia or glucose deprivation [115,116,117]. In addition to NADH and FADH_2_, FA oxidation also produces NADPH via the subsequent processing of the acetyl-CoA entering the Krebs cycle, combining with oxaloacetate to generate citrate, which is then exported to the cytoplasm to produce NADPH catalysed by cytosolic malic enzymes [118]. This enables cancer cells to overcome oxidative stress by providing sufficient NADPH to prevent the accumulation of harmful reactive oxygen species (ROS) [119]. Although several enzymes are involved in FA oxidation, the majority of the research effort has been focused on CPTI, since this is the rate-limiting enzyme for this pathway. 

#### Carnitine Palmitoyltransferase I

Carnitine palmitoyltransferase I (CPTI) converts fatty acyl-CoA to its corresponding fatty-acyl carnitine to be transported into the mitochondrial matrix [120]. Several studies have implicated CPTI in tumorigenesis, with breast [60,61] and ovarian [63] cancers displaying elevated mRNA levels (Table 1). CPTI expression is higher in more aggressive breast cancer subtypes, such as triple-negative breast cancer (TNBC), than in less aggressive ones [61], suggesting a role of CPTI in determining the aggressiveness of gynaecological cancers. Supporting this, CPTI overexpression is associated with poor prognosis, as shown in ovarian cancer patients [63]. Through pharmacological and genetic analysis, CPTI is found to be critical in the progression of breast [62,116], ovarian [63] and endometrial [43] cancer, most likely in an MYC-dependent manner [84]. Lowered CPTI expression in A2780CIS ovarian cancer cells reduced their resistance against cisplatin-induced apoptosis [121], suggesting a role of CPTI in promoting chemoresistance in gynaecological cancers.

## 4. Oestradiol Regulation of Fatty Acid Metabolism in Gynaecological Cancers

In Vitro experiments have demonstrated the crucial role of oestradiol in promoting the aggressiveness of gynaecological cancers by dysregulating FA metabolism. Oestradiol downregulated CD36 in several breast cancer cell lines and promoted their proliferation [38]. In oestradiol-treated breast cancer cells, ACSL4 was implicated in increasing the cellular uptake of polyunsaturated FAs and enhancing cancerous growth [107,122]. Oestradiol is reported to upregulate FASN expression and drive proliferation in an FASN-dependent manner in several gynaecological cancers, including breast [123,124] and endometrial [125] cancer, by far making FASN the most studied FA metabolism enzyme, with regards to its alteration by oestradiol in gynaecological cancers. However, the mechanisms linking oestradiol to the dysregulation of FA metabolism in these cancer types are still underexplored. Nevertheless, several lines of evidence suggest oestradiol could impact the activity of the FA metabolism enzymes through multiple pathways, in addition to its canonical pathway, which are described below in this section.

### 4.1. Oestrogen Receptor Pathway

Oestradiol canonically binds to oestrogen receptors (ER), through which signalling plays a critical role in the development and maintenance of female reproductive function [126], by regulating a myriad of cellular activities, including FA metabolism [127]. Given this role, it is not surprising that the dysregulation of ER is implicated in the progression of gynaecological cancers, notably breast cancer. Activated ER, apart from binding to its response elements as transcription factors, forms multi-protein complexes by recruiting coregulators to dynamically adjust its transcriptional activity, through specific, coregulator-dependent histone modifications [128]. 

**Figure 2:**
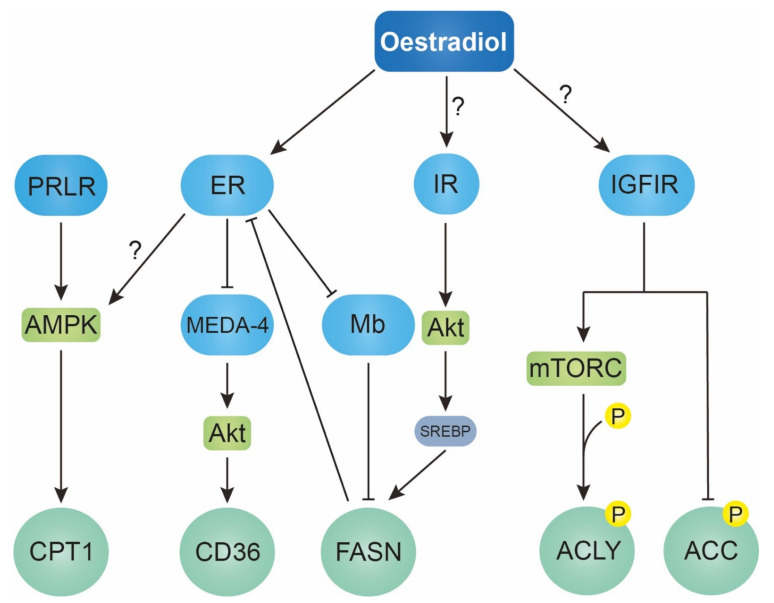
Oestradiol dysregulates the activity of FA metabolism enzymes in gynaecological cancers via the ER and possibly also the IGFIR and IR pathway. Solid lines with an arrowhead represent positive interaction whereas crossbars represent negative interaction. Question marks (?) represent interactions suggested indirectly by current evidence, but more direct evidence is needed to establish their existence. MEDA-4, Mesenteric oestrogen-dependent adipose 4, IGFI, Insulin-like growth factor 1; IGFIR, Insulin-like growth factor 1 receptor; mTORC, Mammalian target of rapamycin complex 1; Mb, Myoglobin; IR, Insulin receptor; ER, Oestrogen receptor; PRLR, Prolactin receptor; AMPK, 5’ adenosine monophosphate-activated protein kinase; SREBP, Sterol-regulatory element binding protein; ACLY, ATP-citrate lyase; ACC, Acetyl-CoA carboxylase; FASN, Fatty acid synthase; ACS, Acyl-CoA synthetase; CPTI, Carnitine palmitoyltransferase I.

Kristiansen et al., found that, in breast cancer tissues, oestradiol suppressed the expression of both ER and non-muscle myoglobin (Mb) [36], a well-known mobile carrier of oxygen, with emerging evidence of its role as an oncogene [129,130]. Mb is also found to strongly co-localise with both ER and FASN, suggesting a potential binding between Mb, with both ER and FASN [36]. Owing to the FA-binding properties of non-muscle Mb [131], oestradiol could enhance intracellular FA availability in gynaecological cancers by disrupting FASN-Mb/ER binding through lowering Mb and ER expression (Figure 2). The increased FA availability allows FASN-produced FAs to be used by the cancer cells instead of being sequestered through Mb/ER binding. Interestingly, in endometrial cancer cell lines, the growth of high-ER-expressing Ishikawa cells was more retarded by FASN inhibition than that of low-ER-expressing HEC1B cells [100]. Thus, the degree to which gynaecological cancers depend on FA synthesis for growth appears to be dependent to oestradiol via ER signalling.

The ER pathway is also known to crosstalk with other signalling pathways, notably the prolactin receptor (PRLR) pathway [132,133]. Prolactin has an important role in lactation but has since expanded to encompass an array of functions, ranging from metabolic homeostasis to maternal behaviour [47]. In gynaecological cancers, prolactin is implicated in ovarian and endometrial cancer, where PRLR is found to be upregulated [134]. Linher-Melville et al., found prolactin stimulated the expression and activity of CPTIA in MDA-MB-231 cells, in an AMPK-dependent manner. Prolactin also partially restored FA oxidation when CPTIA and AMPK were knocked down, demonstrating the crucial role prolactin plays in promoting FA oxidation in breast cancer. Apart from the signalling interaction between ER and PRL pathway, the oestradiol–ER complex has been reported to transcriptionally upregulate both prolactin and PRLR in breast cancer [135]. This evidence describes an indirect way oestradiol could upregulate CPTI and enhance FA oxidation in breast cancer through the ER-PRLR crosstalk and direct upregulation of the PRLR pathway by oestradiol.

Mesenteric oestrogen-dependent adipose (MEDA-4) regulates adipogenesis, adipocyte differentiation and lipid accumulation, serving as a key protein in oestradiol-regulated region-specific fat deposition [136]. Li et al., recently showed MEDA-4 expression negatively correlated with breast cancer patient survival and confirmed the role of MEDA-4 in promoting epithelial-to-mesenchymal transition (EMT), in an AKT-dependent manner, using breast cancer cell lines [137], showing the importance of MEDA-4 in breast cancer pathogenesis. These results, however, were obtained without investigating the role of oestradiol and MEDA-4 in FA metabolism. More relevantly, oestradiol is known to downregulate MEDA-4, most likely through the ER pathway [47,82,90,101,102,103,104,105], subsequently lowering CD36 expression levels in adipocytes [136]. Given oestradiol induces proliferation and downregulates CD36 in several breast cancer cell lines [38], a mechanism similar to this might also be operating in breast cancer (Figure 2), suggesting oestradiol could promote the malignancy of breast cancer in a CD36-dependent manner, by downregulating MEDA-4. Nevertheless, more studies are needed to unravel, in breast cancer pathogenesis, the mechanism linking oestradiol-regulated MEDA-4 and CD36, specifically its FA-uptake role, given its multifaceted properties of being both pro- and anti-carcinogenic, through a multitude of mechanisms [37].

Notably, ER itself can be regulated by ACSL4, as ER expressions were reduced in MCF-7 Tet-Off/ACSL4 and restored upon treatment with doxycycline, suggesting ACSL4 negatively controls ER expression and mechanistically determines ER status in breast cancer along with its dependency on oestradiol [138]. Further, in breast cancer, knocking down FASN dramatically lowered (by >100 fold) the amount of oestradiol needed to activate ER transcriptional activity, and pharmacologically inhibiting FASN in ER-negative breast cancer cells, stably transfected with ER, increased oestradiol-induced ER-mediated transcriptional activity [124]. Therefore, given the potential impact on treatment approach, the possibility of mutual regulation between ER and FA metabolism enzymes should be considered.

### 4.2. Insulin-like Growth 1 Receptor Pathway

Apart from ER, current evidence strongly suggests that oestradiol could also bind to other surface receptors, traditionally associated with other ligands. Yang et al., investigated the effect of oestradiol on the progression of ER- metastatic breast cancer using 4T1 tumour mammary cells. Despite lacking ER, oestradiol-treated 4T1 had greater invasiveness in vitro and metastasised to the lungs faster in vivo [139]. In line with this, a case-control study within the EPIC cohort (European Prospective Investigation into Cancer and Nutrition) found that oestradiol positively correlated with not only ER+ breast cancer types, but also ER- ones (OR = 2.11) [140]. These results suggest that oestradiol could be promoting the carcinogenesis of ER- gynaecological cancers in an ER-independent manner. Indeed, Zhu et al., examined the potential of oestradiol to induce DNA synthesis through the IGFI pathway in uterine epithelial cells. Treatment with IGFIR inhibitor picropodophyllin (PPP) inhibited not only oestradiol-induced DNA synthesis, but also oestradiol-stimulated IGFIR phosphorylation [141]. Interestingly, they also found that oestradiol-stimulated IGFIR signalling on DNA synthesis is independent of the action of oestradiol through ER signalling [141], further suggesting that oestradiol acts as a ligand for IGFIR to instigate IGFIR signalling and its subsequent downstream effects, which could also include those related to FA metabolism. 

IGFIR signalling has been reported to dysregulate FA metabolism in gynaecological cancers, contributing to their development and progression. In a study by Chen et al., IGFI was shown to rapidly induce ACLY Ser-455 phosphorylation, a post-translational modification crucial for regulating lipid metabolism in breast cancer. This phosphorylation promoted glucose-to-lipid conversion, both of which were reduced in the presence of the mTORC1/2 dual inhibitor WYE-132 [77]. Additionally, transfecting various ACL phospho-mutants into MDA-MB-453 inhibited its growth, which was rescued using shRNA-resistant constructs [77]. Overall, this evidence suggests that IGFI promotes the growth of gynaecological cancers, at least in breast cancer. This carcinogenic effect of IGFI is achieved via mTORC-dependent phosphorylation of ACLY Ser-455 to enhance ACLY function for glucose-derived lipid synthesis, probably including FAs too. Furthermore, Balaban et al., found IGFI induced the de-phosphorylation of pACC by inhibiting the physical interaction between pACC and BRCA1. Silencing BRCA1 in MCF7 lowered pACC levels and increased MCF7 proliferation [142], suggesting a IGFI-induced reduction in pACC is involved in retarding the growth of gynaecological cancers. However, other investigators found lowered pACC to be anti-carcinogenic in breast cancer patients treated with metformin, a type 2 diabetes mellitus drug, with accumulating evidence showing its strong potential as an anti-cancer agent [143]. 

Moreover, the ER- breast cancer subtype TNBC was found to not only have higher IGFIR signalling activity than ER+ breast cancers [141], but also exhibited greater FA elongation and uptake [144,145]. More conclusive data on the metabolic differences between ER+ and ER- breast cancers are, however, needed, as these metabolic studies rely on mRNA expression of a panel of metabolic proteins involved in FA metabolism, which do not necessarily reflect overall metabolic activity [146], to infer the metabolic trends of the various breast cancer subtypes. Nevertheless, this evidence strongly suggests oestradiol may also alter the FA metabolism of gynaecological cancers by dysregulating enzymes, such as ACLY and ACC, through the IGFIR signalling pathway, probably by serving as an IGFIR ligand. More studies, however, are needed to determine if direct oestradiol-IGFIR binding does exist and, if so, the specific kinetics of such binding. Nevertheless, it is highly possible for such binding to occur, since oestradiol is found to directly bind to insulin receptors (IR), with which IGFIR shares up to 65% homology in the ligand-binding domain and up to 85% in the tyrosine kinase and substrate recruitment domain [131,147]. In addition, Huang et al., found insulin-stimulated FASN expression in breast cancer cells, with a concomitant increase in SREBP expression and pAkt/Akt ratio. Reducing these with docosahexaenoic acid (DHA) abrogated insulin-stimulated FASN expression, suggesting insulin promotes FASN expression in breast cancer by upregulating SREBP through the Akt pathway [148]. The high degree of homology in IR and IGFIR could also indicate possible activation of IR signalling by oestradiol, further suggesting another non-ER signalling pathway, through which oestradiol may dysregulate FA metabolism in gynaecological cancers. 

There is still much to be investigated as to the exact pathways in which oestradiol, upon binding to ER, dysregulates FA metabolism in gynaecological cancers. Furthermore, emerging evidence strongly indicates oestradiol could influence FA metabolism in these cancer types, through not only the classical ER pathway, but also non-ER pathways, such as the IGIFR pathway. Therefore, subsequent research focus should be expanded to encompass not only investigating the key molecular players of the ER pathway, but possibly those of the non-ER pathways too, such as the IGFIR pathway, in understanding oestradiol-induced FA dysregulation in gynaecological cancers, diversifying the potential candidates to be pharmacologically targeted in treating these cancer types.

## 5. Additional Roles of Oestradiol on Fatty Acid Metabolism in Gynaecological Cancer in the Context of Obesity

Oestradiol level is tightly influenced by obesity, a condition marked by increased adiposity, which alters the profile of bioactive molecules produced, including oestradiol. Notably, oestradiol is synthesised by adipose tissues: adipocyte aromatase converts androgens to oestradiol, and they assume the role of the primary producer of oestradiol post-menopause [149,150]. This adiposity-related elevation of serum oestradiol is compounded by the decreased levels of serum hormone-binding globulin [151], exacerbating the bioavailability of serum oestradiol in obese women. 

Evidently, obesity, whether measured as BMI or long-term weight gain, is linked with the increased risk and poorer prognosis of gynaecological cancers. In breast cancer, every increase in BMI by 5 kg/m^2^ in premenopausal women is associated with an estimated 8% increase in breast cancer risk [152], and a meta-analysis found the relative risk of total mortality for breast cancer in obese women was 1.41 [153], while postmenopausal women who have gained 5–10 kg were at higher risk of breast cancer, with a hazard ratio of 1.16 compared with their stable-weigh counterparts [154]. Similarly, the odd ratio of ovarian cancer risk related to obesity is found to increase for certain subtypes of ovarian cancer, such as borderline serous (1.24 per 5 kg/m^2^) [155], and the risk of mortality from ovarian cancer increases by 3% per 5-unit increase in BMI [156], in line with the increased mortality of 68% in ovarian cancer patients who gained ≥10 lbs from their 20 s to 5 years before their prognosis, compared with those who gained <10 lbs within the same period [157]. The relative risk for endometrial cancer was, staggeringly, 2.89 per 10 units in BMI [158], and endometrial cancer patients face a 9.2% increase in the odds of all-cause mortality per 10% increase in BMI [159], consistent with the increased risk of endometrial cancer by 21% per 5 kg gained since the lowest adult weight [160]. The multivariate odds ratio of cervical cancer risk is 1.70 (1.10–2.63) for obese women [161] and, in a population of women undergoing cervical cancer screening, 20% of cervical cancers could be ascribed to overweightness or obesity [162]. Therefore, oestradiol may be, in part, responsible for the tight epidemiological link between obesity and gynaecological cancers. Indeed, a meta-analysis found the excess risk for breast cancer associated with a 5 kg/m^2^ increase in BMI was lowered from 19% to 2% after adjusting for free oestradiol, a drastic decrease, strongly suggesting that the increased bioavailability of oestradiol is largely responsible for the increased risk of breast cancer in obese women [163].

Although there is increasing evidence describing the role of oestradiol on FA metabolism in gynaecological cancers under non-obese conditions, how oestradiol affects the FA metabolism in obese gynaecological cancer patients remains unclear. There is evidence linking a potential role of oestradiol in obesity-driven pathophysiological pathways, including, inflammation and oxidative stress. Greater understanding of how the FA metabolism-related, pro-carcinogenic action of oestradiol is impacted by these pathophysiological pathways could broaden the approaches in treating these cancer types, through the additional use of anti-inflammatory and antioxidant agents, on top of conventional chemotherapeutic regime, in treating gynaecological cancers, particularly in obese patients.

### 5.1. Inflammatory Pathway

The association of obesity with inflammation has long been recognised, with current evidence pointing to inflammation linking obesity to the pathogenesis of gynaecological cancers. For instance, obese and overweight breast cancer patients who had undergone breast-preserving surgery were observed to have more inflammatory foci or crown-like structures in their mammary adipose tissues compared with their normal-weight counterparts [164], suggesting inflammation being involved in the progression and development of breast cancer in obese patients. Obesity-linked inflammation involves the activation of the NF-κB signalling pathway, where NF-κB is localised to the cytoplasm, where it is bound to a family of inhibitors called inhibitors of κB (IκBs). In response to inflammatory-inducing signals, these inhibitors are phosphorylated by IKK kinase complexes, which direct them to proteasomal degradation, allowing NF-κB to translocate into the nucleus [165]. In the nucleus, NF-κB promotes the gene expression of a host of inflammatory mediators, such as IL-6 and TNF-α [165]. 

Oestradiol levels are known to also be regulated by inflammatory mediators, which can upregulate the expression of aromatase [166,167]. Mechanistic insights linking oestradiol, obesity, inflammation and gynaecological cancer risk through FA metabolism dysregulation could be gleaned from Mauro et al., who examined how adiponectin, an established adipocyte-secreted anti-inflammatory mediator [168] that is inversely related to both BMI [169] and oestradiol levels [170,171], influenced the action of oestradiol in breast cancer. Low levels of adiponectin are found to increase MCF7 viability by promoting the recruitment of LKB1 as a coactivator by ERα, in a dose-dependent manner [169]. This pro-tumorigenic property of adiponectin, particularly at low levels, likely stems from the decoupling of AMPK from LKB1 after recruitment by ERα, allowing the AMPK target ACC to stay active to promote FA synthesis [169]. Therefore, oestradiol-driven dysregulation of FA metabolism through inflammation could be further enhanced in obese breast cancer patients through inflammation, exacerbating their cancer malignancy. However, this enhancement may be restricted based on the presence of ER. Adiponectin-treated MDA-MB-231 xenograft, in contrast to MCF7, had lower tumour volume and higher levels of phosphorylated ACC, suppressing FA synthesis [169]. Highlighted here is the importance of accounting for the specific molecular subtype of gynaecological malignancies in understanding their biology. More relevantly, different molecular subtypes (e.g., ERα+ vs. ERα- breast cancer) can have divergent, possibly opposite, metabolic behaviours, including under pre-existing conditions, such as inflammation during obesity.

### 5.2. Oxidative Stress Pathway

Another pathological component of obesity is oxidative stress, a state of imbalance between the production and elimination of reactive oxygen species (ROS), such as superoxide radicals (O_2_^−^) and hydrogen peroxide (H_2_O_2_) [172]. Though low levels of ROS have physiological functions, accumulation of ROS can lead to pathological consequences, stemming from the ROS-catalysed modifications of important biological macromolecules, such as lipids, proteins and nucleic acids [172]. Oxidative stress has also been suggested to drive the progression of gynaecological cancers in obese patients. Isprostanes, a byproduct of free radical lipid peroxidation of arachidonic acid, was found to be positively linked to breast cancer risk, and this correlation is found to be stronger in obese and overweight patients compared with normal-weight patients [173], suggesting a relationship between obesity, oxidative stress and the pathogenesis of gynaecological cancer. A study by Sateesh et al., however, found no correlation between oxidative stress status and adiposity in breast cancer patients [174], but this apparent lack of correlation may stem from the small sample size of only 30 women, which rendered the study underpowered, prompting the need for a larger sample size before any relationship, or lack thereof, between oxidative stress status, adiposity and breast cancer can be robustly discussed.

The mechanisms underlying the oxidative stress promotion of carcinogenesis is an area of active investigation [175,176], but oestradiol could play a key role in the ROS-mediated promotion of gynaecological cancers. Through a prospective observational study, Madeddu et al., investigated the relationship between BMI and oxidative stress in a population of post-menopausal breast cancer patients. BMI and ROS levels were found to be positively correlated with tumour size, node involvement and distant metastasis in ER+ breast cancer patients [177], suggesting the possible role of oestradiol mediating the pro-carcinogenicity of ROS in gynaecological cancers for obese patients. Furthermore, the oestradiol-generating enzyme sulfatase has been reported to be upregulated in the breast tissue of obese women compared with their normal-weight counterparts. A catalytical thiol group of sulfatases in the active site can be activated by oxidation and may be oxidised by ROS during oxidative stress, activating sulfatase and elevating oestradiol levels [178,179]. Given ROS is also known to dysregulate FA metabolism in cancer cells [180,181], elevation of oestradiol levels by oxidative stress could also, therefore, be a pathway by which FA metabolism could be altered in obese-related gynaecological cancers. 

## 6. Current Update on Therapeutic Strategies Targeting Fatty Acid Metabolism

Clearly, FA metabolism plays a vital role in promoting the development and progression of gynaecological cancers. Therefore, enzymes involved in FA metabolism are attractive targets in treating these cancer types. Moreover, inhibiting these enzymes can synergistically augment the antitumour effects of chemotherapeutic agents targeting the oestradiol pathway (e.g., selective ER modulators (SERM) and aromatase inhibitors) or to overcome chemotherapeutic resistance against these agents in gynaecological cancers. In addition to the developing pharmacological inhibitors specifically targeting FA metabolism enzymes, interest is also growing in implementing diet-based intervention to supplement conventional chemotherapeutic regime.

### 6.1. CD36 Inhibition

FA uptake is critical in supplying cancers with exogenous FAs for their progression; therefore, targeting the FA transporter CD36 could represent a promising strategy for treating some of these gynaecological cancers. One type of recently studied CD36 inhibitors, known as thrombospondin-1 (TSP-1) mimetic peptides, mimics the structure of CD36 ligands. Although many TSP-1 mimetics have been developed and studied (reviewed in [182]), three were found to have antitumour activity in gynaecological cancers: ABT-510, ABT-526 and ABT898 (Table 2). ABT510 is derived from the second properdin type I repeat of the NH2-terminal third of TSP-1 and, in mouse models, it inhibited the growth of epithelial ovarian cancer, as well as increasing its susceptibility to chemotherapeutic drugs [183,184]. Despite its initial promise as a CD36 inhibitor to treat ovarian cancer, it was thereafter abandoned, as phase II clinical trials of ABT510 treatment in advanced renal cell carcinoma [185], soft tissue sarcoma [186] and metastatic melanoma [187] showed it lacked sufficient clinical efficiency. ABT-526 is a GVITRIR heptapeptide based on the second TSP-1 type 1 repeat. It exhibited antitumour activity in dogs bearing the metastasis of mammary carcinoma, with one dog being relapse-free following ABT-526 treatment [188]. More promising are second-generation TSP-1 mimics, such as ABT898, which are more stable and better tolerated [186]. ABT898 regressed established ovarian tumours in animal models and significantly prolonged disease-free survival compared with control animals [189]. Although it is established that the anti-tumorigenic properties of these TSP-1 mimics stem from the ensuing anti-angiogenic effect upon CD36 binding, the inhibition of the FA uptake role of CD36 could also play a role in this regard. Indeed, the binding of TSP-1 to CD36 is known to inhibit the uptake of the long-chain FA myristic acid in a nitric-oxide-dependent manner [190]. The uptake of other long-chain FAs, such as palmitic acid by CD36, could also be inhibited by TSP-1, operating in parallel with the anti-angiogenic effects of TSP-1 in curbing the growth of gynaecological cancers.

Recent studies have implicated CD36 in promoting the resistance of breast cancer towards tamoxifen, a widely used selective ER modulator for treating ER+ breast cancer. Liang et al., found that the CD36 protein expression was higher in tamoxifen-resistant MCF-7 (MCF7/TAMR) than their non-resistant counterpart, suggesting the role of CD36 in mediating tamoxifen resistance in MCF7/TAMR [39]. Indeed, knocking down CD36 in MCF7/TAMR via siRNA restored sensitivity towards tamoxifen, as evidenced by tamoxifen regaining the ability to inhibit the growth of MCF7/TAMR. These results poInt. to a treatment strategy where ER+ breast cancer patients are administered simultaneously with tamoxifen and CD36 inhibitors to overcome tamoxifen resistance. This strategy, however, may not be effective in all tamoxifen-resistance breast cancer types, since in the same study, Liang et al., found MDA-MB-231, an ER-negative tamoxifen-resistant breast cancer cell line, had lower CD36 protein expression than non-resistant MCF7 [39]. Therefore, utilising the tamoxifen-CD36 inhibitor combination might require stratifying breast cancer patients based not only on tamoxifen resistance, but also on both CD36 and ER expression levels. 

### 6.2. ATP-Citrate Lyase (ACLY) Inhibition

Numerous natural and synthetic ACLY inhibitors are available (reviewed in [205]) but only a few have been tested in gynaecological cancers. Hydroxycitrate (HCA) was found to reduce the cancer stem cell population of mammary breast cancer cell lines (HMLE and HMLER), suggesting it may reduce tumour initiation [191]. Besides, metformin, together with caffeic acid, reduced the protein level of ACLY in cervical carcinoma SiHa/HTB-35 cells, impairing FA synthesis and sensitising SiHa/HTB-35 to the action of cisplatin (Table 2) [192]. This combination effect suggests ACLY could have a role modulating the response of cancer cells towards certain chemotherapeutic agents, at least in cervical cancer. Interestingly, inhibiting ACLY reduced the intracellular citrate level and cell viability of breast cancer cell lines more effectively than inhibiting citrate transport protein (CTP) [206], but whether inhibiting both CTP and ACLY produces a synergistic antitumour effect is underexamined. Targeting ACLY, however, may not be a viable long-term treatment option, as cancer cells can upregulate acetyl-CoA synthetase (ACCS) to produce acetyl-CoA from acetate, obviating the need for citrate as a source of acetyl-CoA for FA synthesis [207]. This bypassing could be overcome by inhibiting ACLY together with ACCS simultaneously, but whether this approach results in favourable clinical outcomes needs further investigation. 

Few studies have investigated the effect of ACLY inhibitors in influencing the effect of tamoxifen on breast cancer cells. Ismail et al., found that co-treating MCF-7 with HCA and tamoxifen reduced MCF-7 viability and promoted apoptosis to a greater degree than when either one was used alone [208], suggesting their antitumour effects are acting synergistically when used in combination. This evidence could prompt a clinical trial, investigating whether the synergistic effect of ACLY inhibitors and tamoxifen can be translated clinically in breast cancer patients, which, if successful, could motivate lowering the therapeutic dosage of tamoxifen required, so as to lessen the side-effects undergone by tamoxifen-treated patients [209]. Apart from that, other investigators have demonstrated the potential use of targeting ACLY in overcoming drug resistance in several cancer types, such as hepatocellular carcinoma [210] and ovarian cancer [211]. Therefore, it would also be interesting to investigate the potential of using the tamoxifen-ACLY inhibitor combination in overcoming tamoxifen-resistance in breast cancer. Nevertheless, the possibility of the breast cancer cells gaining resistance towards this tamoxifen-ACLY inhibitor combination should be borne in mind, since Ismail et al., found elevated levels of triglycerides in the co-treated MCF-7 [208], suggesting a compensatory mechanism to acquire FAs, without the need for citrate-originating acetyl-CoA. 

### 6.3. Acetyl-CoA Carboxylase (ACC) Inhibition

ACC inhibitors, such as TOFA, are found to retard the growth of breast [193] and ovarian [45] cancer, but none have reached the clinical trial phase for the treatment of gynaecological cancers (Table 2). However, the ACC inhibitor NDI-010976 may be investigated as a potential gynaecological cancer treatment. It reduced de novo lipogenesis in overweight adult male subjects in a randomized, double-blind, crossover study [212]. Although inhibiting ACC may have clinical benefits in treating gynaecological cancers, this method should be approached with caution, as several tumour types have been reported to have their growth accelerated when ACC is inhibited [213]. 

So far, the role of the ACC inhibitor and its impact on the pro-carcinogenic activity of oestradiol has received little attention, probably stemming from the focus on FASN instead of ACC as the key enzyme to target FA synthesis in cancer. Nevertheless, studies suggest ACC may be a valuable additional target in treating breast cancer patients undergoing treatment with aromatase inhibitors. Du et al., deprived SUM44, an invasive lobular breast cancer cell line, of oestrogen long term to mimic aromatase inhibition and also treated these long-term oestrogen-deprived cells (SUM44 LTED) with TOFA to inhibit ACC [214]. They found, compared with the parental controls, TOFA more greatly inhibited the cell growth of SUM44 LTED, suggesting the potential use of ACC inhibitors to supplement the aromatase treatment of breast cancer. Nevertheless, the effectiveness of the aromatase inhibitor-ACC inhibitor combination might be influenced by the Human Leucocyte Antigen (HLA) typing of the invasive lobular breast cancer, given that Du et al., also found the cytotoxic effect of TOFA was not enhanced in MM134 LTED, another invasive lobular breast cancer cell line but of a different HLA typing from SUM44 [215], compared with their parental controls [214].

### 6.4. Fatty Acid Synthase (FASN) Inhibition

Pharmacologic FASN inhibitors are classified based on the FASN domain targeted, that is, whether they target the β-ketoacyl synthase or thioesterase domain [216]. Orlistat, a well-studied irreversible inhibitor of the thioesterase domain, is shown to exhibit antitumour properties in various breast and ovarian cancer cell lines (Table 2) [194]. It is also a well-established anti-obesogenic agent, shown to reduce weight by about 3% in obese and overweight people compared with their placebo counterparts [217]. This weight-losing effect suggests another mechanism through which orlistat may lower the risk of gynaecological cancers, in concert with its FASN inhibitory activity. Natural FASN inhibitors are also available, the most studied of which is epigallocatechin gallate (EGCG), shown to inhibit the growth of breast cancer cells in vivo and in vitro [195,196]. Intriguingly, selective FASN inhibitor Fasnall operates by targeting co-factor binding to FASN, not by competing with substrate intermediate of FASN. It had potent anticancer activity in various breast cancer cell lines and in MMTV-Neu in vivo model of HER2^+^ breast cancer, with favourable pharmacokinetics and tolerance profiles [197]. Notably, it had a synergistic effect on tumour shrinkage when combined with carboplatin. Such synergistic effects suggest Fasnall can be used together with carboplatin in the clinical treatment of breast cancer to improve their efficacy [197]. TVB-2640 is a first-in-class FA inhibitor used in a Phase I trial to investigate its efficiency in lowering metabolic markers associated with non-alcoholic fatty liver disease in obese men [218]. TVB-2640 is part of an ongoing phase II clinical trial that seeks to determine how effective it is in combination with paclitaxel and trastuzumab in treating ER2+ breast cancer metastases [198]. C75 is a synthetic FASN inhibitor found to exert antitumour effects in breast [82,101], ovarian [219] and endometrial [48] cancer. Given it demonstrated anti-carcinogenic properties in a broad range of gynaecological cancers, it could be a suitable candidate drug for clinical trials investigating its efficacy and effectiveness in these cancer types.

Interestingly, inhibition of FASN in gynaecological cancers by agents, such as cerulenin and C75, also impairs oestradiol-induced nuclear accumulation of ER and downregulates ER expression [219], reinforcing the antitumour effects of FASN inhibition by diminishing the pro-tumorigenic signalling emanating through the ER pathway, which could also reduce the impact of oestradiol-induced upregulation of FASN [220]. Coupling FASN inhibitors to inhibitors of aromatase, the enzyme converting androgens into oestrogens, could serve as potential therapeutic strategy in aromatase inhibitor-treated ER+ breast cancer patients to recurrence, which is due to the ability for aromatase inhibitors, such as anastrozole, to upregulate ER-dependent FASN protein expression in this cancer type by inhibiting ubiquitin-mediated FASN protein degradation [221].

### 6.5. Acyl-CoA Synthetase (ACS) Inhibition

Owing to its importance in permitting FAs to be utilised for both catabolic and anabolic downstream processing, blocking FA activation by inhibiting ACS might also serve as an effective approach to treat gynaecological cancers. However, few drugs have been developed to target ACS, a situation most likely arisen due to the existence of numerous ACS isoforms, which might necessitate separate drugs to be specifically made for each isoform. Nevertheless, drugs found to be specific for certain ACS isoforms do exist, such as thiazolidinediones, a drug targeting PPARγ for the treatment of type 2 diabetes and involved in potent-specific inhibition of ACSL4 [222]. Therefore, thiazolidinediones could be potential therapeutic agents in the treatment of breast cancer, a cancer type in which ACSL4 was found to play a role promoting its malignancy and chemoresistance. Investigators also found inhibiting ACSL4 in the presence of chemotherapeutic drugs can have a synergistic antitumour effect, demonstrated by treating MDA-MB-231 with triacsin C alongside cisplatin, doxorubicin or paclitaxel (Table 2) [111]. Additionally, other non-cancer drugs may be repurposed for targeting ACS in cancer cells, such as aspirin, which was found to suppress the abnormal lipid metabolism of HCC cells through inhibiting acyl-CoA synthetase long-chain family member 1 (ACSL1) [223]. This, however, may not be clinically beneficial in treating breast cancers, as a population-based study demonstrated that long-term use of low-dose aspirin marginally increased the risk of breast cancer [224], but this does not preclude its potential in treating other gynaecological cancers.

Among the myriad ACS isoforms, ACSL4 has been given the most attention so far for inhibition studies, given the mounting evidence on its pro-carcinogenic role. Indeed, Wu et al., investigated the role of ACSL4 in promoting resistance against tamoxifen in breast cancer. Specifically, they found overexpressing ACSL4 reduces the cytotoxic effect of tamoxifen in MCF-7 and SKBr-3, suggesting inhibiting ACSL4 could overcome tamoxifen resistance in breast cancer [54]. Indeed, through ERα inverse agonist XCT-790 and triacsin C co-treatment, Dattilo et al., found the co-treatment synergistically reduced the proliferation of MDA-MB-231 [201]. However, XCT-790 also acts as a mitochondrial uncoupler, independent of its ERα-related activity, possibly being responsible instead for its synergism with triacsin C in breast cancer [225]. Which activity mode of XCT-790 is acting synergistically with triacsin C needs to be ascertained, as it could impact whether this treatment combination can be administered to ER-negative breast cancer patients.

### 6.6. Carnitine Palmitoyltransferase (CPTI) Inhibition

FA oxidation provides cancer cells the ATP and NADPH needed to support their uncontrollable proliferation, making this process an attractive target for gynaecological cancer therapy. Few CPTI inhibitors are available, namely, perhexiline [203], etomoxir [62,84,202], and Eugenol [44], all of which showed antitumorigenic effect when used to treat breast cancer in vitro and in vivo (Table 2). Although etomoxir is not clinically approved for any gynaecological cancers, investigators found chemically inhibiting CPTI with etomoxir, together with glutaminase with CB-839, a drug currently in Phase I/II, decreased cell proliferation and migration of CB-839-resistant TNBC cells more than inhibiting only either enzyme alone [202]. This effective dual combination may open up a possibility of conducting a clinical trial to investigate whether etomoxir and CB-839 could be used simultaneously to improve the treatment of aggressive breast cancer. Nevertheless, it should be borne in mind that etomoxir might not affect all cancer types the same way. Some cell lines of several cancer types, including MCF7 and HeLa, did not experience any reduction in proliferation when treated with etomoxir [226]. Furthermore, etomoxir was also found to exhibit an off-target effect with complex I of the electron transport chain being targeted at high doses of etomoxir [226]. This unintended consequence should be considered when determining the appropriate dose for clinical trials and cancer studies, should its intended target be only CPTI. Recently, a reversible CPTI inhibitor ST1326 was found to reduce the proliferation of chronic lymphocytic leukaemia cells [227] and could be considered for targeting CPTI in gynaecological cancers. Eugenol, as one of the components of a topical antiviral spray AV2, was found to be marginally more effective in regressing HPV-associated precancerous lesions of the cervix in a low-resource setting Phase III clinical trial, though the regression rate did not achieve statistical significance. This resource-restricted outcome may be rectified with a larger sample size and by repeating the clinical trial in a high-resource setting [204].

Similar to most other enzymes involved in FA metabolism, little is known about how CPTI could influence the pro-tumorigenic properties of oestradiol. Nevertheless, current evidence implicates CPTI in modulating the resistance of breast cancer against SERM. Duan et al., found that MCF7/TAMR had higher basal expression of CPTI than parental controls and their CPTI expression became higher than their respective baseline when both were treated with endoxifen, another SERM developed to address tamoxifen-resistant breast cancer [228]. Furthermore, in both untreated and endoxifen-treated MCF7/TAMR, inhibiting AMPK via compound C and siRNA knockdown lowered CPTI expression, while, in contrast, the expression was increased when inhibiting AKT with MK2206. These results suggest CPTI could be involved in promoting SERM resistance in breast cancer, which is positively and negatively regulated by the AMPK and AKT pathway, respectively. However, these results need to be confirmed by determining whether SERM-resistance in untreated and endoxifen-treated MCF7/TAMR could be abrogated by knocking down or inhibiting CPTI.

### 6.7. Omega-3 Fatty Acids Supplementation

Aside from pharmacological interventions, dietary intervention is worth exploring, specifically in supplementing and, therefore, enhancing well-tested cancer therapies. Omega-3 FAs, such as eicosapentaenoic and docosahexaenoic acids (EPA and DHA, respectively) are among the nutrients heavily studied for their potential use in this approach. Indeed, consumption of omega-3 FAs is associated in numerous studies with the decreased risk of multiple cancer types, including gynaecological cancers [229,230], accomplished primarily through their anti-inflammatory action [231]. Indeed, the use of omega-3 FAs to inhibit the progression of gynaecological cancer may be more beneficial in obese patients, as rats given high-fat diets had lower NF-κB mRNA levels and DNA binding than control-diet rats when both were treated with omega-3 FAs [232]. Other than their anti-inflammatory properties, omega-3 FAs could also retard the growth of gynaecological cancers by interfering with the action of oestradiol, directly or indirectly, in dysregulating FA metabolism in these cancers. Huang et al., found DHA abrogated the FASN upregulation and pAkt/Akt increase in MCF7 induced by oestradiol, while also inhibiting oestradiol-induced promotion of the SREBP isoform SREBP-1 protein expression and these abrogations were further enhanced upon adding the Akt inhibitor LY294002 [148]. Pro-tumorigenic impact of oestradiol on FASN in breast cancer could, therefore, be significantly inhibited with DHA supplementation in combination with Akt-inhibiting agents (Table 2). However, it remains uncertain whether, in addition to SREBP-1, the abrogation of oestradiol-induced FASN by DHA could also be, in part, due to the effect of DHA on PPARs, since PPARs are also known to be both activated by DHA or its downstream metabolites [233], and involved in regulating FA metabolism, including FA synthesis [234]. Nevertheless, intake of omega-3 FAs, however, should be accompanied with caution, as excess consumption is linked to several adverse effects, including the increased risk of prostate cancer [235], another hormone-responsive cancer type, which may also imply the elevated risk of gynaecological cancers. Therefore, optimising the omega-3 FAs intake of each patient is paramount, to avoid such side effects when administering omega-3 FAs, alone or together with established cancer treatments, to fully take advantage of the tremendous therapeutic potential of omega-3 FAs [12].

## 7. Future Directions

Despite great advancement in understanding the role of oestradiol in driving the development and progression of gynaecological cancers by dysregulating FA metabolism, there are areas where we still have a poor understanding. First, most in vitro and clinical studies investigating FA metabolism in gynaecological cancers based their conclusions related to the state of FA metabolism in these cancer types on either the mRNA or protein expression of enzymes involved in FA metabolism. Though mRNA and protein expression do correlate with protein activity, trends in protein activity can dramatically diverge from those of its mRNA or protein expression, as mRNAs can be regulated post-transcriptionally and proteins post-translationally [236]. More direct evidence of enzymatic activity is needed before robust conclusions on the FA metabolic phenotype of gynaecological cancers can be made. Second, though the treatment of oestradiol has been shown to alter FA metabolism in gynaecological cancer cells, evidence demonstrating the signalling pathway mediating this process is still severely lacking, prompting the need to not only further elucidate the molecular details of how activating the classical ER pathway by oestradiol dysregulates FA metabolism in gynaecological cancers, but also to uncover non-ER receptors, to which oestradiol could bind and activate, considering evidence of oestradiol being capable of directly binding to IR [147] or activating the IGFIR pathway independent of ER [141]. This understanding allows not only the identification of more diverse potential drug targets to inhibit the pro-carcinogenic action of oestradiol, but also the prediction of chemotherapeutic resistance against SERM, as chemotherapeutic blockage of a signalling pathway can be circumvented by cancer cells by using alternative pathways [237]. Third, research focus in this area has been narrowly focused on breast cancer and should be broadened to encompass the other gynaecological cancers, given all of them have intimate physiological commonalities by virtue of their responsiveness to female sex hormones, notably oestradiol. Crucially, acquiring this understanding encompassing all the gynaecological cancers will enable the identification of potentially shared vulnerabilities in FA metabolism, which can be clinically exploited to develop broad-based therapies in treating these cancer types.

## 8. Conclusions

FA metabolism in gynaecological cancers is a multi-faceted process, involving uptake, de novo synthesis, activation and oxidation. Up to now, our understanding of how oestradiol dysregulates FA metabolism in the pathogenesis of these cancer types is still in its infancy. In this review, we have comprehensively reviewed the dysregulation of key FA metabolism enzymes in gynaecological cancers and how oestradiol could play a part in this dysregulation by activating the ER and IGFIR pathways. We also briefly discussed how the increased levels of oestradiol, secondary to inflammation and oxidative stress under obese conditions, may alter FA metabolism, affecting the progression of gynaecological cancers. In addition, several promising drugs rectifying dysregulated FA metabolism are now at various stages of development and these can be potentially combined with oestradiol pathway-targeting chemotherapeutic agents to enhance the potency of, or to circumvent, cancer resistance against these agents. The therapeutic use of omega-3 FAs is a significant milestone in applying dietary interventions, as an adjunct to well-established regimes in the treatment of gynaecological cancers.

## Figures and Tables

**Table 1:** Summary of the various fatty acid metabolism enzymes and their effects on the phenotypes of gynaecological cancers.

Enzyme	Cancer Type	Expression Level	Cancer-Associated Phenotype	Selected References
CD36	Breast	↑	Increased proliferation Increased migration	[34]
	Ovarian	↑	Enhanced metastasis	[31]
	Cervical	↑	Enhanced metastasis	[35]
	Endometrial	N/A	N/A	N/A
ACLY	Breast	↑	Enhanced metastasis	[40]
	Ovarian	↑	Increased proliferation	[41]
	Cervical	↑	Enhanced metastasis	[42]
	Endometrial	↑	N/A	[43]
ACC	Breast	↑	Increased proliferation	[44]
	Ovarian	↑	Enhanced tumour growth	[45]
	Cervical	N/A	N/A	N/A
	Endometrial	↑	N/A	[43]
FASN	Breast	HER2+-↑ Luminal A, TNBC-↓	Enhanced metastasis	[46,47]
	Ovarian	↑	Increased tumour growth	[48,49,50,51]
	Cervical	↑	N/A	[52,53]
	Endometrial	↑	N/A	[43]
ACS	Breast	QNBC-ACSL4 ↑, ER (-)-ACSL1↑, ACSL3 ↑, ACSL4 ↑, ACSL5 ↑	Increased migration Increased invasion	[54,55,56,57,58]
	Ovarian	ASCL3 ↓, ASCL 5↓	Increased proliferation	[58]
	Cervical	N/A	N/A	N/A
	Endometrial	ACS5 ↓	N/A	[59]
CPTI	Breast	↑	Enhanced metastasis	[60,61,62]
	Ovarian	↑	Increased proliferation	[63]
	Cervical	N/A	N/A	N/A
	Endometrial	N/A	N/A	N/A

ACLY, ATP-citrate lyase; ACC, acetyl-CoA carboxylase; FASN, Fatty acid synthase; ACS, Acyl-CoA synthetase; CPTI, Carnitine palmitoyltransferase I.

**Table 2:** A non-exhaustive list of interventions for the main FA metabolism enzymes at various stages of development.

Target Protein	Intervention	Cancer Type	Preclinical Model	Clinical Trial	References
CD36	ABT-526	Breast	Breast cancer-bearing dogs	-	[188]
	ABT898	Ovarian	Xenografts	-	[189]
ACLY	Hydroxycitrate	Breast	In Vitro	-	[191]
	Metformin	Cervical	In Vitro	-	[192]
ACC	TOFA	Ovarian, Breast	Xenografts	-	[45,193]
FASN	Orlistat	Breast, Ovarian	Xenografts	-	[194]
	Rigallocatechin Gallate	Breast	Xenografts	-	[195,196]
	Fasnall	Breast	Xenografts	-	[197]
	TVB-2640	Breast	-	Phase II	[198]
	TVB-3166	Ovarian	Xenografts	-	[199]
	C93	Ovarian	Xenografts	-	[200]
	DHA Supplementation	Breast	In Vitro	-	[148]
ACS	Triacsin C	Breast	In Vitro	-	[111,201]
CPTI	Etomoxir	Breast	Xenografts	-	[202]
	Perhexiline	Breast	Xenografts	-	[203]
	Eugenol	Breast	In Vitro	-	[44]
		Cervical	-	Phase III	[204]

ACLY, ATP-citrate lyase; ACC, acetyl-CoA carboxylase; FASN, Fatty acid synthase; ACS, Acyl-CoA synthetase; CPTI, Carnitine palmitoyl transferase I; DHA, Docosahexaenoic acid.
